# Supplementary material for: The Expression and Secretion Profile of TRAP5 Isoforms in Gaucher Disease
Source: Cells. 2024 Apr 20;13(8):716. doi: 10.3390/cells13080716 (PMC11049511; doi:10.3390/cells13080716)
Supplement: Supplementary file 1 [file cells-13-00716-s001.zip › cells-2903949-supplementary.pdf]

cells

Supplementary Materials

The Expression and Secretion Profile of TRAP5 Isoforms in Gaucher Disease

Supplemental Table 1. Demographics, genotypes, and clinical characteristics of bone disease in patients with GD at the time of the study partially have been previously published [18, 21]. GD, Gaucher disease; ERT, enzyme replacement therapy; SRT, substrate reduction therapy; EM, Erlenmeyer; AVN, avascular necrosis.

nt No.	Age yrs.	Sex M/F	ERT/SRT duration (yrs.)	Genotype	Chito nmol/hr/mL	Lyso Gb-1 ng/mL	CCL18 ng/mL	Bone Pain	Bone marrow infiltration	EM-flask deformity	Pathologic fractures/ Bone surgery	AVN
**No bone complication. Total 11**												
P1	50	F	ERT 5-10	N370S/N370S	52	2.7	60.9	No	Yes	Yes	No/No	No
P2	25	F	ERT >10	L444P/L444P	273	15.9	180.4	No	No	No	No/No	No
P3	18	F	ERT 5-10	N370S/F213I	215	7.9	54.5	Yes	Yes	Yes	No/No	No
P4	41	F	SRT	N370S/R463C	160	10.9	159.4	No	Yes	Yes	No/No	Yes
P5	29	F	SRT	N370S/N370S	619	6.7	75	No	No	No	No/No	No
P6	56	F	ERT >10	N370S/N370S	40	1.1	41	Yes	Yes	Yes	No/No	Yes
P7	37	F	ERT >10	N370S/W381X	133	NA	41	Yes	Yes	No	No/No	Yes
P8	49	F	ERT >10	N370S/L444P	44	4	68	Yes	Yes	No	No/Yes	Yes
P9	29	F	ERT 5-10	/L444P	34	7	105	No	No	No	No/No	No
P10	21	M	SRT	N370S/L444P	228	13.3	76	No	Yes	No	No/No	No
P11	33	M	ERT 1-5	R48Q/L444P	1022	11	1087.9	Yes	Yes	Yes	No/No	No
**Osteopenia (OSN). Total 14**												
P12	29	F	ERT 1-5	N370S/N370S	1104	29.6	170.1	No	Yes	Yes	No/Yes	No
P13	61	F	ERT >10	N370S/N370S	103	2.5	210.2	Yes	Yes	Yes	Yes/No	Yes
P14	32	F	ERT 5-10	N370S/L444P	127	10.7	63.1	No	No	No	No/No	No
P15	52	F	SRT	N370S/N370S	42	1	37.3	No	Yes	No	No/No	No
P16	40	F	SRT	N370S/R463C	5	1	79.1	Yes	Yes	No	No/No	No
P17	41	F	naive	N370S/N370S	1194	49	407	No	No	No	Yes/No	No
P18	51	F	SRT 5-10	N370S/L444P	314	11.7	289.2	No	No	No	No/No	No
P19	74	F	SRT 1-5	N370S/N370S	283	1.4	191	Yes	Yes	Yes	No/No	No
P20	38	F	ERT >10	N370S/N370S	110	35	276	Yes	No	No	No/No	No
P21	61	F	1 year	N/A	2080	375	662	Yes	No	No	No/No	No
P22	65	M	SRT 5-10	N370/N370	44	1.5	56.1	Yes	Yes	No	No/No	No
P23	18	M	naive	N370S/N370S	164	5.1	97.5	No	Yes	No	No/No	No
P24	37	M	ERT 1-5	N370S/ V394L	493	12	103	Yes	No	No	Yes	No
P25	20	M	ERT >10	L444P/P266A	118	7.5	58.2	No	No	No	No/No	No
**Osteoporosis (OSR) Total 14**												
P26	57	F	SRT 5-10	L444P/R502C	118	3.4	95.5	Yes	Yes	Yes	No/No	No
P27	54	F	ERT >10	N370S/L444P	663	129.5	131.4	Yes	Yes	Yes	No/No	Yes
P28	20	F	ERT 1-5	N370S/L444P	665	36	145.5	Yes	No	No	No/No	No
P29	62	F	SRT	N370S/N370S	7	1	61.2	Yes	Yes	Yes	No/No	No
P30	30	F	ERT >10	N370S/N370S	116	17.6	120.1	No	Yes	No	No/No	No
P31	63	F	naive	N370S/R496H	38	2.5	45.7	Yes	Yes	Yes	No/No	No
P32	44	F	ERT/SRT >10	L444P/R493C	530	42.7	178.5	Yes	Yes	No	Yes/Yes	No

P33	68	F	ERT 5-10	N370S/R463C	63	2.5	123.8	Yes	No	Yes	No/Yes	Yes
P34	45	F	ERT 5-10	N370S/N370S	19.96	5.4	13.8	Yes	No	Yes	No/Yes	No
P35	46	F	SRT	N370S/N370S	97.8	39	331	Yes	No	Yes	No	Yes
P36	55	M	SRT 0-1	N370S/L444P	376	15.2	120.2	Yes	No	No	Yes/Yes	No
P37	36	M	ERT >10	N370S/Y412X	546	85.6	562.9	Yes	Yes	Yes	Yes/Yes	Yes
P38	39	M	ERT 5-10	N370S/N370S	57	39.5	836.5	No	No	No	No/No	No
P39	42	M	SRT 5-10	N370S/N370S	254	35.8	171	No	Yes	No	No/Yes	No

Supplemental Table 2. The sequences of gene primers that have been used for real-time PCR.

Gene	Primers Sequence	
GAPDH	Forward	GAGAGGGCTGGGCTCATTTGCAG
	Reverse	CCATCCACAGTCTTCTGGGTGGCAG
CCL2/MCP-1	Forward	GCAATCAATGCCCCAGTCA
	Reverse	TGCTGCTGGTGATTCTTCTATAGCT
ACP5	Forward	CGTATTCTCTGACCGCTCCC
	Reverse	TCTTGAAGTGCAGGCGGTAG

Supplemental Table 3. Prevalence of osteopenia and osteoporosis in female and male patients with Gaucher disease by age group. NB – normal bone mineral density, OSN – osteopenia, OSR – osteoporosis.

	Female			Males		
	NB	OSN	OSR	NB	OSN	OSR
Patients number	9	10	10	2	4	4
Minimum age (Years)	18	29	20	21	18	36
Maximum age (Years)	56	74	68	33	65	55
Mean age (Years)	37	48	49	27	35	43
Std. Deviation	13	14	15	8.5	22	8.4
Std. Error of Mean	4.3	4.6	4.8	6.0	11	4.2
